# Target-distractor competition cannot be resolved across a saccade

**DOI:** 10.1038/s41598-018-34120-4

**Published:** 2018-10-24

**Authors:** Kiki Arkesteijn, Jeroen B. J. Smeets, Mieke Donk, Artem V. Belopolsky

**Affiliations:** 1 Department of Experimental and Applied Psychology, Vrije Universiteit, Amsterdam, The Netherlands; 2 Department of Human Movement Sciences, Vrije Universiteit, Amsterdam, The Netherlands

## Abstract

When a distractor is presented in close spatial proximity to a target, a saccade tends to land in between the two objects rather than on the target. This robust phenomenon (also referred to as the global effect) is thought to reflect unresolved competition between target and distractor. It is unclear whether this landing bias persists across saccades since a saccade displaces the retinotopic representations of target and distractor. In the present study participants made successive saccades towards two saccadic targets which were presented simultaneously with an irrelevant distractor in close proximity to the second saccade target. The second saccade was either visually-guided or memory-guided. For the memory-guided trials, the second saccade showed a landing bias towards the location of the distractor, despite the disappearance of the distractor after the first saccade. In contrast, for the visually-guided trials, the bias was corrected and the landing bias was eliminated, even for saccades with the shortest intersaccadic intervals. This suggests that the biased saccade plan was remapped across the first saccade. Therefore, we conclude that the target-distractor competition was not resolved across a saccade, but can be resolved based on visual information that is available after a saccade.

## Introduction

We make about three saccades every second to direct our fovea to objects of interest. At any moment in time, multiple objects in the visual scene can compete for selection. Since visual information in our brain is represented in retinotopic (eye-centered) coordinates, every saccade dramatically changes the visual input. This makes it challenging to keep track of relevant and irrelevant objects across saccades^1^. In the present study we investigated how competition between relevant and irrelevant objects in the scene is resolved across saccades.

It is well-known that distracting elements can compete with saccadic targets and alter saccade planning. For example, the eyes are often first directed to an irrelevant salient abrupt onset instead of a target^2,3^. Even when the eyes are successfully directed to the target, the influence of a distractor is also evident from longer saccadic latencies (i.e., “the remote distractor effect”^4^) or alterations in saccade trajectory, such that saccades curve either towards or away from the distractor location^5,6^. Saccade curvature towards the distractor location is primarily observed for short-latency saccades and is thought to result from unresolved competition, while curvature away is typically associated with long latency saccades and is thought to reflect inhibition of the distractor location^7^.

The presence of a distractor does not only influence the shape of the saccade trajectory but can also affect saccade endpoints. When a distractor is presented in close spatial proximity to a target, the saccade plan is biased towards the distractor and this leads to a saccade landing in between the two objects, which is generally known as the global effect (or saccade averaging)^8–10^. The global effect can be modulated by the relative size of the two objects, with saccades landing closer to the larger object^9^. In addition, the global effect is affected by the difference in direction between the target and distractor, with a maximal global effect occurring with a difference of around 20–30°^11^. Moreover, the size of the global effect reduces with a higher target location probability^12^ and depends on saccade latency, such that it becomes smaller with increasing saccade latency^9,13,14^. To explain the latter results, it has been suggested that with increasing latency, observers are better able to fully process the target location and as a result, saccades become progressively more accurate^15^. An important question is whether the global effect also occurs in the presence of an intervening saccade which would require a remapping of the planned saccade based on information about target and distractor acquired before the first saccade. If target-distractor competition is not resolved before the execution of the first saccade, the planned second saccade could be biased by the distractor.

Only a few studies have explored how a saccade plan is remapped across a saccade. An efficient updating mechanism would entail a rapid emergence of spatiotopic representations of distractors and targets directly after the saccade. This has been observed in a study by Jonikaitis & Belopolsky^16^, in which participants were asked to make a sequence consisting of a horizontal and a vertical saccade. They showed that an abrupt onset distractor that was presented and extinguished right before the start of this sequence made the second saccade curve away from its spatiotopic location. This suggests that the plan for the second saccade was biased by the presentation of the distractor before the start of saccade sequence and was remapped across the first saccade (for similar results see van Leeuwen & Belopolsky^17^; Boon *et al*.^18^).

A second example of visual contextual effects that were robust for an intervening saccade was obtained by de Brouwer and colleagues^19^. In their study, participants were presented with a Brentano version of the Müller-Lyer illusion and had to make a memory-guided saccade from one endpoint of the illusion to the middle vertex. The illusion changed the perceived distance between fixation and the vertex as indicated by biased saccade landing positions. Importantly, a similar bias in the landing positions was observed even when a preceding saccade in a different direction was introduced. However, a single saccade from the intervening saccade target to the vertex was not influenced by the illusion, suggesting that the perceived direction between the first and second targets of the double-step saccade sequence was not affected by the illusion. de Brouwer and colleagues^19^ concluded that the illusion biased the saccade plan made before the start of the saccade sequence and that biased plan was updated across the first saccade.

In apparent contrast with the results discussed in the previous paragraph, Silvis and colleagues^20^ showed that target-distractor competition was resolved (the global effect eliminated) when a small intervening saccade in the direction opposite to the final saccade destination was introduced. In their study participants made a sequence of two saccades, while both target and distractor were continuously present during the whole saccade sequence. Both objects were visible before and after the first saccade, therefore it remains unclear if target-distractor competition was resolved across the saccade, or if the second saccade plan was based solely on information available after the first saccade. In the present study we explored what information about the locations of target and distractor is updated across saccades.

The goal of the present study was to investigate whether the target-distractor competition is resolved across saccades. Specifically, we were interested in how information available before and after the first saccade is used to create and update the saccade plan of the second saccade. To achieve this, we manipulated the availability of target and distractor information across saccades, eliciting both visually-guided and memory-guided saccades. The global effect is traditionally considered reflexive in nature and almost solely measured with visually-guided saccades, while both stimuli remain visible until saccade execution^8,9,21^. Furthermore, it is known that saccades to remembered locations tend to be less accurate^22^. For these reasons, to examine the target-distractor competition, we measure the change in landing position as a result of distractor location (“the landing bias”). The landing bias is measured as a difference in saccade landing position when a distractor is presented left from the target compared to when a distractor is presented right from the target. This method controls for intrinsic and extrinsic noise in landing position and has previously been used in studies investigating saccade curvature^16,17^. Additionally, we examine the time-course of resolving the target-distractor competition across a saccade. This was done in two separate experiments.

## Experiment 1

Participants performed a sequence of two successive target-directed saccades, one horizontal and one vertical. Target-distractor competition was induced by presenting a distractor close to the second saccade target. The distractor was always removed before the eyes landed on the first saccade target. In one condition (visually-guided), the second saccade target remained on the screen. In the other condition (memory-guided), the second saccade target disappeared with the distractor, resulting in a memory-guided second saccade. If the presence of the second target after the first saccade is enough to adjust the saccade plan then the landing bias should be reduced to zero in the visually-guided condition. Alternatively, the biased saccade plan could persist, similar to the results obtained from saccade curvature^16^. In this case, an unresolved competition will manifest itself in the second saccade being biased towards the distractor. Adjusting the saccade plan should be especially difficult in the memory-guided condition, which should be evident in a considerable landing bias for the second saccade. By looking at the time-course, we examined how the saccade plan is adjusted in time. Following earlier results by Heeman *et al*.^13^, we expect the size of the landing bias to become smaller following longer intersaccadic intervals (i.e., the time between landing of the first saccade and start of the second saccade).

### Methods

#### Participants

Eighteen students (aged 18–34, 8 women), including one of the authors, of the Vrije Universiteit Amsterdam took part in the experiment. Two of them were excluded from the analysis, because more than 40% of the trials were rejected (criteria explained below). Participants received either money (8€ per hour) or curriculum credits as compensation for their time. All had normal or corrected-to-normal vision and were naive to the purpose of the study. An informed consent was obtained from all participants and both Experiment 1 and 2 were conducted with approval by the Ethical Committee of the Faculty of Behavioral and Movement Sciences of the Vrije Universiteit Amsterdam and all rules, regulations and guidelines were followed.

#### Apparatus

The experiment was conducted in a dimly lit room. The stimuli were presented on a 21‘’ LCD monitor (Samsung 2233RZ) with a 1680 × 1050 pixel resolution and a 120 Hz refresh rate. Gaze was recorded using the Eyelink 1000 (SR research) with a temporal resolution of 1 ms and a spatial resolution of 0.01°. The experimental software controlling the stimulus presentation, response collection and eye tracking was written with OpenSesame version 2.9^23^ using a PsychoPy back-end^24^ and PyGaze^25^. An automatic algorithm detected saccades using minimum velocity and acceleration criteria of 35 °/s and 9,500°/s^2^.

#### Stimuli and procedure

**Figure 1:**
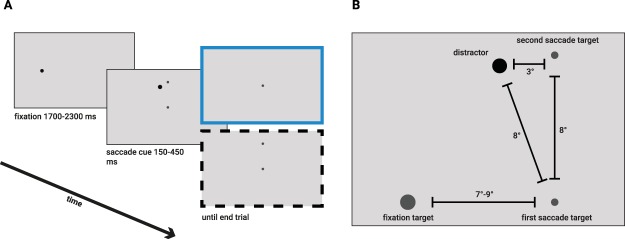
An example of the trial sequence in Experiment 1. (**A**) Each trial started with participants fixating the fixation point. After a delay, a distractor and two saccade targets were presented simultaneously. Participants were asked to make a rapid saccade sequence, consisting of a horizontal and a vertical saccade. In the memory-guided condition (top, blue frame) both the second saccade target and the distractor were removed during the first saccade. In the visually-guided condition (bottom, black dashed frame) only the distractor was removed during the first saccade while the second target remained present. (**B**) Scaled spatial lay-out of the stimuli. One of the four configurations that were used in Experiments 1 and 2 is shown: the right-up saccade sequence.

Participants were seated with their head positioned on a chin and forehead-rest at a viewing distance of 75 cm from the display. Stimuli were presented on a light grey (76 cd/m^2^) background (Fig. 1). The trial began with a dark grey (13 cd/m^2^, radius = 0.2°) fixation dot that was positioned either left or right from the center of the screen (center-fixation distance was randomly chosen between 7° and 9° in steps of 0.22°). After a variable time interval (drawn from a Gaussian distribution, μ = 2000 ms, σ = 300 ms) two saccadic targets (dark grey 13 cd/m^2^, radius = 0.2°) and one distractor (black: 0 cd/m^2^, radius = 0.4°) were presented^9,26^. Participants were instructed to make a rapid sequence of a horizontal and vertical saccade towards the two saccade targets (Fig. 1). We used four different saccade sequences (right-up, right-down, left-up and left-down) and 10 different amplitudes for the first saccade to make sure participants were looking at the stimuli rather than repeating the same movement.

The first target was always presented in the center of the screen and the second target was presented 8° up or below the first target. The distractor was presented at 7.42° vertically (either on the top or the bottom half of the screen, corresponding to the second target position) and 3° horizontally left or right from the first saccade target. This ensured that the Euclidean distance from the first target to the distractor and to the second saccade target was the same (8°). In the visually-guided condition, the distractor disappeared as soon as the saccade towards the first target was detected. In the memory-guided condition, both the second saccade target and distractor disappeared as soon as the first saccade was detected. Participants received auditory feedback if the first saccade did not land within 2° from the first target or the second saccade did not land within 3° from the second target. In addition, if participants made a saccade faster than 80 ms or slower than 450 ms, they received a visual warning.

We presented trials in blocks that contained each combination of starting direction (left, right), second target location (up, down), distractor position (left, right) and condition (visually-guided, memory-guided), resulting in 16 unique trials per block. Each block contained 32 trials. The experiment was done in one session of 60 minutes (this included calibration and practice trials), which resulted in 15–17 experimental blocks (the number of blocks was dependent on time) and 480–544 trials per participant.

### Data analysis

Eye-tracking data were analyzed offline. Saccades were detected using the automatic detection by the Eyelink system. We used a custom written code (Python) to extract all relevant details and events. The ‘first saccade’ was defined as the first saccade which started after the targets were displayed that ended within 2° of the first target. The second saccade was defined as the first saccade which started from the first target that ended within 4° of the second target. Trials in which the saccade landing positions fell out of the defined boundaries were excluded. Furthermore, trials in which the first saccade latency was shorter than 80 ms or longer than 450 ms were excluded from analysis. Trials in which participants had an intersaccadic interval that was shorter than 50 ms or longer than 600 ms were also excluded. For two participants this resulted in a loss of more than 40% of the trials. These participants were excluded from further analysis. For the remaining 16 participants 9% of the trials were dismissed on the basis of saccade landing position boundaries and 10% on the basis of saccade latency criteria. Note that corrective micro-saccades after the first saccade were ignored if they started and ended within 4° of the first target. This was the case for 8% of the remaining trials. 81% of all trials were used for further analysis.

**Figure 2:**
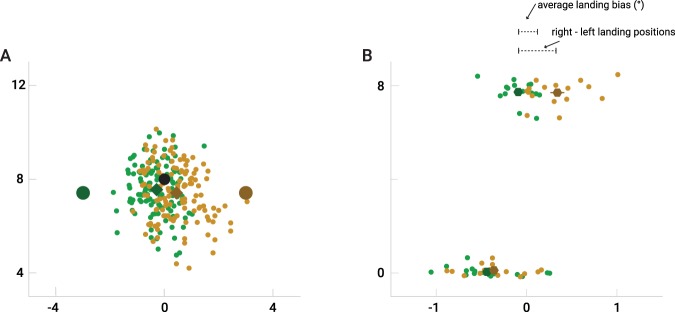
Example of saccade landing positions of Experiment 1. (**A**) All landing positions taken from the memory-guided condition from one participant when the distractor was presented left (green) and right (yellow). (**B**) Mean saccade landing-positions for both first and second saccade for all participants in the memory-guided condition (same color coding as in A). Note the different scaling for the horizontal and vertical direction in panel B.

The trajectories from trials of each of the four saccade sequences were rotated and/or mirrored to the same reference frame: starting direction from left, second saccade up. We examined the influence of the distractor on the second saccade endpoints by calculating the landing bias: the signed horizontal difference between saccade endpoints when the distractor was presented to the right (positive sign) of the target to when the distractor was presented to the left of the target (negative sign), divided by 2 (see Fig. 2). These calculations were done using values in degree of arc. Since right minus left difference was taken, the landing bias would be positive if the saccade landed in between target and distractor and the landing bias would be negative if saccade landed away from distractor and target. If there were no effect of distractor presence, the landing bias should be zero. In contrast, if there was a maximum effect of distractor presence (i.e., if saccade landed at the distractor instead of the target), the landing bias should be 3 degrees. If saccade landed away from where the distractor was presented (having the same horizontal deviation as the distractor but at the other side), then the landing bias should be −3. We tested whether the landing bias differed from zero in each of the conditions by a one-sample t-test.

To get a precise estimate of the time-course of the landing bias as a function of intersaccadic interval, we smoothed the saccade endpoints (For a detailed description^17,27–29^) using a moving Gaussian window (step size 1 ms and σ = 20 ms). A smoothed landing position time series was made for each condition and difference in distractor location per each participant, and then averaged across all participants for intersaccadic intervals 150–400 ms, this time window was based on the distribution of the intersaccadic intervals. To asses if there was a landing bias the difference in saccade landing position was tested against zero using a one sample t-test for each step of the Gaussian smoothed time series.

A linear regression was fit to the smoothed time series for both conditions for every participant. To determine if the size of the landing bias became smaller over time, a one sample t-test against zero was performed on the resulting slopes.

For all descriptive statistics we report the means and standard deviation across participants.

### Results

The latency of the first saccade was on average 174 ± 24 ms. This saccade undershot the first target by about 5%: the mean horizontal landing position was −0.4°. This first saccade had a 0.04 ± 0.08° landing bias (Fig. 2B).

**Figure 3:**
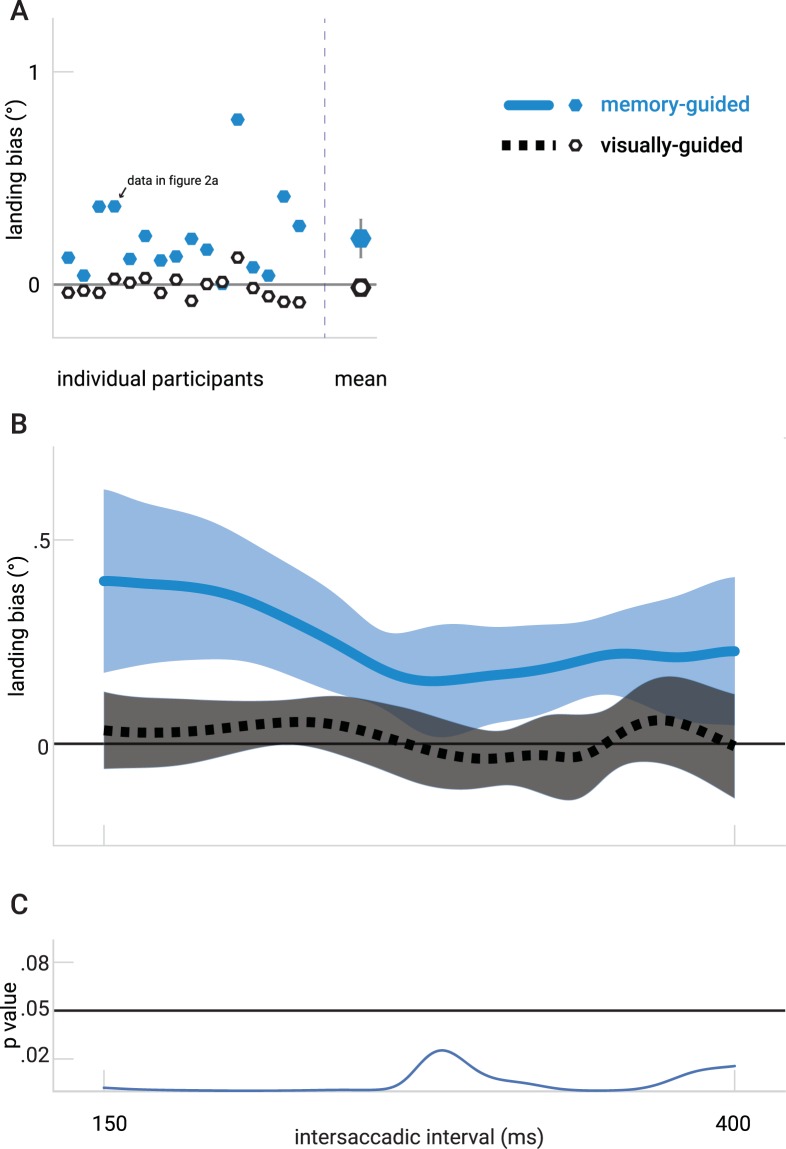
The results of Experiment 1. (**A**) Left from the vertical dashed line: Mean saccade landing position of the second saccade plotted per participant as the difference (in degrees of visual angle) between when the distractor was presented left or right for the visually-guided (black) and memory-guided (blue) conditions. Right from the vertical dashed line: Mean landing position difference across all participants (error bars reflect 95% within-subjects confidence interval, note that for the visually-guided condition the error bars are ‘hidden’ in the diamond) (**B**) The time-course of the landing bias in Experiment 1. The two curves depict the Gaussian smoothed average difference of saccade endpoints between the two distractor positions for the two conditions. The transparent areas indicate 95% confidence intervals of this difference. (**C**) p-values corresponding to a one sample t-test for each interval for the memory-guided condition.

For the second saccade, every participant showed a positive landing bias (a shift in landing-positions towards the location of the distractor) in the memory-guided condition, but not in the visually-guided condition (Fig. 3A). The landing bias indeed differed significantly from zero in the memory-guided condition (0.22°, t(15) = 4.473, *p* < 0.001) but not in the visually-guided condition (0.01°, t(15) = 1.06, *p* = 0.846). In the memory-guided condition the horizontal landing positions for the distractor left and right trials were respectively: −0.09° ± 0.18° and 0.34° ± 0.30°. In the visually-guided condition the horizontal landing position for the distractor left and right were respectively: 0.01° ± 0.08° and −0.02° ± 0.09°, see Fig. 3B.

The time-course of the landing bias in both visually-guided and memory-guided conditions was analyzed separately, see Fig. 3C. The Gaussian smoothed time series remains constant around zero for the visually-guided condition but seems to decrease for larger intersaccadic intervals for the memory-guided condition. The one sample t-test for every time point revealed that the landing bias was significant larger than zero (p < 0.05) for the whole range of intersaccadic intervals in the memory-guided condition. The landing bias did not differ significantly from zero in the visually-guided condition. The slopes resulting from the linear regression for every participant were not significantly different from zero, both for the memory-guided condition (−0.0018 ± 0.0046 °/ms; t(15) = −1.49, *p* = 0.078) as well as for the visually-guided condition (−0.0002 ± 0.0023 °/ms; t(15) = −0.48, *p* = 0.639).

### Discussion

The present results show that target-distractor competition resulting in a biased saccade plan was preserved across a saccade when the second saccade was memory-guided but was eliminated when the second saccade was visually-guided. This elimination of the landing bias in the visually-guided condition was already evident at the shortest intersaccadic intervals (about 150 ms). This suggests that the second saccade plan which was biased towards the distractor could be very quickly adjusted when the second target remained present after the first saccade. When the second saccade target disappeared, the saccade plan could not be adjusted, as the landing bias was different from zero.

The landing bias in the memory-guided condition tended to become smaller over time, but this decrease was not significant. This lack of significance may be due to the examined time-course being limited to the intersaccadic intervals that occurred naturally (up to 400 ms). It might, therefore, be possible that the landing bias will reduce to zero for longer intersaccadic intervals. If a biased saccade plan can be adjusted over time then the landing bias should reduce to zero during the longer intersaccadic intervals. This could be accomplished, for example, by boosting the representation of the second target from memory. However, if the availability of visual information about the target is a prerequisite for adjusting the direction of the saccade, then a considerable landing bias will remain present even at the longer time intervals. To explore this issue, we experimentally varied the time between the first and second saccade in Experiment 2.

## Experiment 2

The goal of Experiment 2 was to explore whether the landing bias, in the double-step saccade paradigm with the disappearance of the second target, would also remain for intersaccadic intervals longer than 400 ms. To achieve this, Experiment 2 consisted only of the memory-guided condition of Experiment 1. In order to extend the intersaccadic interval, the task was modified such that the execution of the second saccade could be delayed. Specifically, on two-thirds of all trials, after completing the first saccade, participants had to keep fixating the first saccade target until a go-signal was presented, instructing them to execute the second saccade. On the remaining one-third of all trials, participants were allowed to execute the double saccade sequence as fast and accurate as possible as in Experiment 1.

### Methods

The methods are the same as in Experiment 1, except for the aspects mentioned below.

#### Participants

Nineteen students (aged 20–30, 8 women) of the Vrije Universiteit Amsterdam took part in the experiment; two also participated in Experiment 1.One participant was excluded for further analysis based on the same criteria as in Experiment 1. An informed consent was obtained from all participants.

#### Stimuli, design and procedure

The experiment was similar to Experiment 1 with a few important exceptions. First, in Experiment 2 the second saccade target always disappeared together with the distractor after initiation of the first saccade. On one-third of the trials the fixation dot was colored blue (5 cd/m^2^), which indicated to the participants that they should perform the sequence consisting of a horizontal and a vertical saccade as fast and as accurately as possible (no delay condition). On the remaining two-third of all trials, a delay was introduced. On these trials, the fixation was colored dark grey (as in the previous experiment). The participants were told to execute a fast and accurate saccade towards the first target and to maintain fixation on it until it turned blue. This varied per trial between 50–450 ms (in steps of 50 ms) from when the first saccade towards the first target was detected online. When the first saccade target turned into a blue fixation target (as described earlier), it served as go-signal for execution of the second saccade (delay condition). We presented trials in blocks that contained each combination of starting direction (left, right), second target location (up, down), distractor position (left, right) and delay-condition (ratio 2:1; delay: no-delay). This resulted in 24 trials per block and 17–20 blocks (number of blocks was dependent on time) per participant, resulting in a total of 408–480 trials per participant. The experiment was completed in one session of 60 minutes (this included calibration and practice trials).

#### Data analysis

Eye-tracking data were analyzed offline. We used the same inclusion criteria as in Experiment 1, except that we changed the maximum intersaccadic interval from 600 ms to 800 ms. Because the delay and no-delay condition were mixed within one block, we observed that the intersaccadic intervals for both delay and no delay condition were shifted towards longer intervals. Therefore, we decided to combine the trials from these conditions for further analysis. 6% of the trials were excluded based on the basis of saccade landing position boundaries and 13% of the trails were excluded on the basis of saccade latency criteria. 81% of all trials were used for further analysis. The definition of the first and second saccade was the same as in Experiment 1.

The calculations done to determine the landing bias over all trials were the same as in Experiment 1 with one exception: based on the average intersaccadic intervals distributions of every participant, the time window that was used for the time series analysis ranged from 250–600 ms.

#### Results

**Figure 4:**
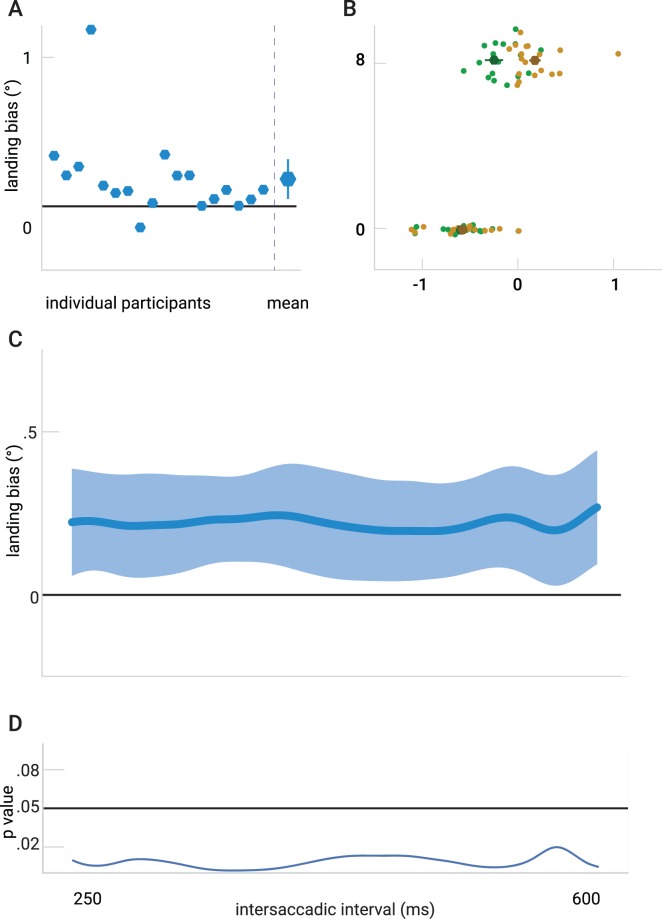
The results of Experiment 2. (**A**) Left: Mean saccade landing position of the second saccade. plotted per participant the difference between when the distractor was presented left or right. Right: Mean saccade endpoint difference across all participants (error bars reflect 95% within-subjects confidence interval). (**B**) Mean saccade landing-positions for both first and second saccade for all participants when the distractor was presented left (green) and right (yellow). (**C**) The time-course of the landing bias in Experiment 2. The curve depicts the average difference of saccade endpoints between the two distractor positions. The transparent area indicates a 95% confidence interval of this difference. (**D**) p-values corresponding to a one sample t-test for each time point of the Gaussian smoothed time series.

The latencies of the first saccades were 174 ± 29 ms. The first saccades undershot the first target by about 7%: the mean landing position was −0.59° ± 0.28°, with a 0.02° ± 0.04° landing bias, see Fig. 4B.

The landing bias was positive for most participants (Fig. 4A); on average it differed significantly from zero (t(17) = 2.78, *p* = 0.006). This replicates the finding of Experiment 1 and suggests that the landing bias persists with longer intersaccadic intervals. The horizontal landing positions for the distractor left and right trials were respectively: −0.25° ± 0.40° and 0.18° ± 0.26°, see Fig. 4B.

We determined the time-course of the landing bias (Fig. 4C) and performed a t-test for each time point (Fig. 4D). We found that for the whole range, the landing bias was larger than zero, p < 0.05. The slopes resulting from the linear regression for every participant were not significantly different from zero (0.0003°/ms ± 0.0011°/ms; t(17) = 1.17, *p* = 0.433).

### Discussion

The findings of Experiment 2 replicate and extend the results observed in Experiment 1. As in the previous experiment, we found a significant difference in landing position of about 0.25° which was preserved across a saccade when both target and distractor disappeared before the end of the first saccade. In addition, the results demonstrate that this difference did not show any sign of decrease over time. Even though the landing bias reduced to zero already for the shortest intersaccadic intervals when the second saccade was visually-guided (Experiment 1), the results of Experiment 2 suggest that the saccade plan could not be adjusted in the absence of visual target information.

## General Discussion

The present results show that target-distractor competition is preserved across saccades. In two experiments using a double-step saccade task, we have demonstrated that the landing bias resulting from target-distractor competition remained larger than zero even after an intervening saccade was made. Importantly, this was only the case when neither the second target nor the distractor was present beyond the first saccade. Surprisingly, we found that even with extended intersaccadic intervals the landing bias did not diminish in size. However, when the second target remained present throughout the trial, the landing bias was reduced to zero. This suggests that observers can only resolve the target-distractor competition when target information is provided after the first saccade. Interestingly, the reduction of the landing bias in the presence of the target was evident already at the shortest time intervals after the first saccade (about 150 ms).

We found a considerable landing bias at the shortest time intervals when the second target disappeared. Remarkably, a detailed time-course analysis of our experiments demonstrated that, in contrast with previous studies^13,15^, the landing bias did not reduce over time. Coeffe & O’Regan^15^ were among the first to show that the saccade latency modulates the size of the landing bias or ‘ the global effect’. In their study, they manipulated the saccade onset time with a delay at fixation. They found a global effect for saccades executed as fast as possible (around 200 ms), yet for saccades executed later (around 350 ms) the global effect was eliminated. Likewise, in a more recent study, Heeman *et al*.^13^ found comparable latency effects on the size of the global effect. They found that there was a considerable landing bias for shorter saccade latencies (220 ms) but it reduced to zero in a linear fashion when saccade latencies became longer (up to 340 ms). In the present study, the global effect remained stable for intervals up to 600 ms, suggesting that competition between target and distractor stops at saccade onset and that the biased saccade plan cannot be adjusted without visual information.

The present results are in line with previous work^16,17^, that demonstrated that the oculomotor system rapidly updates target and distractor information across saccades. The aforementioned studies showed that distractors can alter saccadic trajectories, such that saccades curve away from a distractor, even when it is presented before the intervening eye movement. Here we extend these findings by showing that distractors can also affect future saccadic endpoints. Both previous findings and the results obtained in our present experiments indicate that the saccade metrics of a second saccade depend on information obtained before the first saccade: a biased saccade plan.

Remarkably, in our experiments, we found that when the target remained present, information about its location was rapidly used to correct the biased saccade plan. This is in contrast with the findings of Jonikaitis & Belopolsky^16^ where curvature of the saccade trajectory was still found when the distractor disappeared but the target remained present after the first saccade. This suggests that correction of the biased saccade plan based on the visible target location can be applied only to the distractors that are presented in close proximity to the target, as for the landing bias. However, when the distractor is presented far from the target, the biased saccade plan cannot be corrected early in time and leads to a curved saccade trajectory.

The results obtained from the visually-guided condition complement an earlier study by Silvis *et al*.^20^ who found a landing bias of zero when an intervening saccade in the direction opposite to the final saccade destination was made. In their study, both target and distractor information was available throughout the saccade sequence, and it was suggested that the competition between target and distractor was resolved after the execution of the first saccade. The memory-guided condition in our experiments clearly demonstrated that the biased saccade plan remained beyond the first saccade in the absence of visual information. Only upon landing on the first target, this plan could be quickly adjusted using available visual target information in the visually-guided condition.

The landing bias in our study closely resembles the ‘classical’ global effect that is defined as the tendency of the eyes to move towards an intermediate position^9,13,15^. Interestingly, similar conclusion was reached by Herwig *et al*.^30^ who studied the effect of target-distractor competition on the endpoint of memory-guided saccades and found that their results mimicked the classical global effect. Furthermore, similar to the current results, they argued that it is impossible to resolve the target-distractor competition during the memory interval suggesting that the target and distractor locations are coded together for memory-guided saccades. Similarly, de Brouwer and colleagues^31^ who investigated the effect of the Muller-Lyer illusion on the amplitude of saccadic eye movements, found that the illusion affected the endpoints of the saccades and that the size of this effect was mediated only by the presentation time of the visual stimuli and not by the latency of the saccade or the saccade type (visual-guided or memory-guided). The results from these studies and our experiments indicate that saccades remain biased irrespective of saccade type or even time. The only aspect that seems to play a role in resolving the target-distractor competition is the time that the visual stimuli are presented on the screen. Although top-down control can reduce the size of the global effect^12^, target-distractor competition cannot be completely resolved by time or top-down control when no visual information is available.

It has been argued that attended and unattended locations are updated across saccades and this can lead to attentional facilitation at the future retinotopic location^1,32,33^. In other words, there are predictions about the upcoming visual scene that are formed before a saccade is initiated. In terms of our research, this implies that the saccade plan for the second saccade is formed based on information that is accumulated before execution of the first saccade. In our experiments, this leads to a biased saccade plan. Furthermore, in our design, the predictions about the visual scene after a saccade are not met due to the removal of the distractor during the first saccade. In the visually-guided condition, the presence of the second saccade target allows the oculomotor system to quickly correct the biased saccade plan in the direction of the target. However, in the memory-guided condition, there is no visual information available, thus leaving the oculomotor system with only the information derived before the start of the saccade sequence. This, in turn, results in a biased saccade plan that is immune to memory-based modification.

Previous studies have found predictive responses to an impending saccade in the superior colliculus (SC)^34,35^. The results of our memory-guided condition indicate that competition is updated spatiotopically across saccades. This suggests that competitive activations between the saccade goal and distractor at the SC^7^ did not subside over time thus indicating that neuronal activity at the SC saccade map of both target and distractor was remapped across a saccade.

To summarize, in the present study we show that the landing bias is preserved across saccades. However, visual information about the target is sufficient to adjust the biased saccade plan that was formed by presenting the distractor before the start of the first saccade. Furthermore, when no visual information is available, the existing saccade plan cannot be modified and this results in a target-distractor competition that remains unresolved. The results of this set of experiments are in accordance with the idea that information about stimuli is constantly remapped across saccades and this, in turn, influences the metrics of our movements.
